# Current Research Progress on Long Noncoding RNAs Associated with Hepatocellular Carcinoma

**DOI:** 10.1155/2019/1534607

**Published:** 2019-06-24

**Authors:** Haihong Shi, Yuxin Xu, Xin Yi, Dandan Fang, Xia Hou

**Affiliations:** ^1^Department of Biochemistry and Molecular Biology, Jiamusi University School of Basic Medicine, Jiamusi, Heilongjiang 154007, China; ^2^Jiamusi Maternal and Child Health Hospital, Jiamusi, Heilongjiang 154001, China; ^3^Hospital of Traditional Chinese Medicine of Qiqihar, Qiqihar, Heilongjiang 161000, China; ^4^Department of Physiology, Wayne State University School of Medicine, Detroit, MI 48201, USA

## Abstract

Hepatocellular carcinoma (HCC) is the second leading cause of mortality among cancers. It has been found that long noncoding RNAs (lncRNAs) are involved in many human cancers, including liver cancer. It has been identified that carcinogenic and tumor-suppressing lncRNAs are associated with complex processes in liver cancer. These lncRNAs may participate in a variety of pathological and biological activities, such as cell proliferation, apoptosis, invasion, and metastasis. Here, we review the regulation and function of lncRNA in liver cancer and evaluate the potential of lncRNA as a new goal for liver cancer.

## 1. Introduction

Hepatocellular carcinoma (HCC), the most common form of liver cancer, is involved in 90% of primary liver cancers. In the last few decades, liver cancer has become the most important commonly diagnosed tumor type worldwide. It is also considered to be the most lethal cancer, related to approximately 34% of all malignancies [1, 2]. HCC is a highly invasive and fatal type of tumor that is often involved in relapse and metastasis, and the prognosis is poor. The incidence of liver cancer is related to a variety of risk factors, such as hepatitis B virus (HBV) and hepatitis C virus (HCV) infections, alcoholic cirrhosis, smoking, and aflatoxin B1 intake [3]. However, the molecular mechanism of the occurrence and development of liver cancer is complicated, which is related to different processes, e.g., cell cycle dysregulation, apoptosis, tumor cell invasion, and metastasis [4]. Accumulating proofs show that long noncoding RNA (lncRNA) expression is altered in liver cancer and involved in tumorigenesis [5].

**Figure 1:**
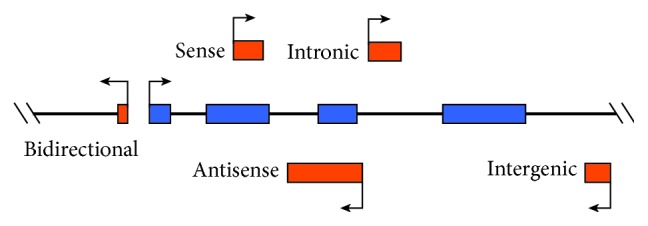
Based on the location of the lncRNA on the genome, it can be divided into five types: (1) sense, (2) antisense, (3) bidirectional, (4) intronic, and (5) intergenic. The coding RNA and noncoding RNA exons are shown in blue and red, respectively.

**Figure 2:**
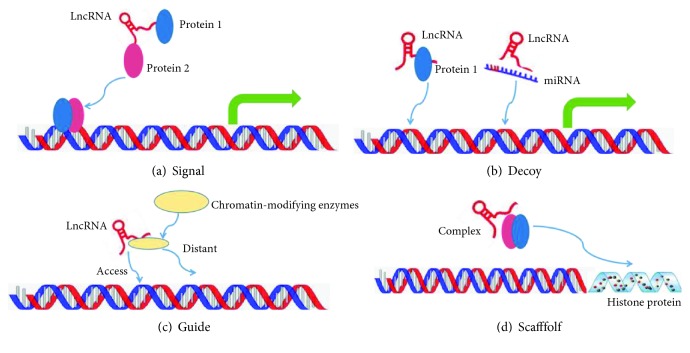
Four typical molecular functions of lncRNA: (a) LncRNAs can be used as molecular signalling mediators to regulate the expression of certain genes together with specific transcription factors or chromatin modifiers. (b) LncRNAs can bind to and titrate the expression of proteins or RNA, which indirectly play a variety of biological functions. (c) LncRNAs recruit chromatin-modifying enzymes that can enter or leave the target gene. (d) LncRNAs can pool multiple proteins to form ribonucleoprotein complexes and affect histone modifications.

In recent decades, most researches concerning the relationship between tumorigenesis and human genes have focused on structural genes and their related regulatory sequences. However, some studies show that noncoding sequences of human genes play crucial roles in tumorigenesis. The human genome contains approximately 3 billion base pairs, of which less than 2% encode proteins, whereas the remaining ~98% of the genome consists of non-protein-coding sequences. RNAs that cannot be translated into proteins are called noncoding RNAs (ncRNAs), which include lncRNA, siRNA, miRNA, and other types [6]. LncRNA consists of more than 200 nucleotides without protein-coding potential but with gene regulatory functions. LncRNAs are classified based on the related locations of their protein-encoding genes in the genome, including (1) sense, (2) antisense, (3) bidirectional, (4) intronic, and (5) intergenic (Figure 1). This positional relationship is helpful in predicting the lncRNA function. LncRNA is involved in the proliferation, migration, invasion, apoptosis, angiogenesis, and drug resistance of tumor cells, although it was previously considered as “transcriptional noise” [7–9]. LncRNA is also related to the regulation of biological functions and gene expression under physiological and pathological conditions [10]. However, only a few functional lncRNAs have been well characterized to date; several mechanistic topics of lncRNA function have been reviewed elsewhere [11, 12]. In this review, a summary of the four well-known molecular functions of lncRNAs is shown (Figure 2): (1) signal function—lncRNA has the function of stimulating the combination of transcription factors, suggesting that it may act as a signal molecule regulating the expression of genes; (2) decoy function—transcription of a class of lncRNAs can then bind to and titrate protein or RNA targets without performing any other functions. (these lncRNAs may negatively regulate the expression of their targets by acting as molecular bait); (3) manipulation function—lncRNAs can recruit the chromatin-modifying enzyme to regulate or control the positioning of these enzymes close to or away from the target gene; and (4) scaffolding function—lncRNAs can aggregate with many proteins to form a nuclear protein complex, which is involved in the modification of histones. LncRNAs have been proven to play an essential biological role in carcinogenesis by regulating gene expression, such as carcinogenic and antitumor functions. LncRNAs can be used as a criterion for the diagnosis and prognosis of hepatocellular carcinoma [13–16].

In this review, we mainly discuss HCC-associated lncRNAs. Here, we summarized the differences in the expression of lncRNAs in HCC; then, we reviewed the participation of lncRNAs in HCC cell proliferation, apoptosis, and migration. Finally, we discussed the prospect of lncRNAs as potential biomarkers and therapeutic targets for HCC.

## 2. Molecular Mechanisms of LncRNAs in HCC

LncRNA is involved in the development of various molecular mechanisms of hepatocellular carcinoma: epigenetic regulation, the regulation of DNA damage and cell cycle progression, microRNA regulation, signal transduction pathways, and hormone-induced cancer [17]. In liver cancer, lncRNAs are used as transcriptional regulatory molecules for oncogenes and tumor suppressor genes [17]. For instance, lncRNA HOTAIR overexpression is associated with the development of liver cancer. In contrast, the lncRNA TARID can prevent cancer formation by causing the demethylation of tumor suppressor gene promoters [18]. Epigenetic regulation refers to genetic phenotypes and genetic changes in gene expression, including DNA methylation, histone modification, and chromatin remodelling, and does not result in any changes in DNA sequencing. Studies show that a crucial role in liver cancer development is that lncRNAs are accomplished by epigenetic regulation. LncRNAs can regulate gene expression through epigenetic regulation, transcriptional and posttranscriptional regulation, etc. LncRNAs participate in various biological processes in liver cancer cells, such as proliferation, differentiation, and apoptosis [19]. Besides, lncRNAs are involved in the regulation of a variety of epigenetic complexes, thus resulting in the activation and inactivation of genes. For example, lncRNA TCF7 is highly expressed in HCC cells and plays an important role in the maintenance of the self-renewal ability of the liver cancer stem cell [20]. The TCF7 gene is activated by recruiting SWI/SNF complexes to the gene and produces lncRNA TCF7, which can activate the Wnt signalling pathway and lead to the occurrence of hepatocarcinoma. The repair of DNA damage and the regulation of cell cycle checkpoints are important to maintain cell integrity. LncRNAs are also involved in DNA damage repair and in the regulation of physiological or pathological processes such as cell cycle, through which lncRNAs can regulate the occurrence and development of tumors [20]. The p53 gene, a tumor suppressor gene, also owns a robust ability to encode transcription factors in cells, which is at a low expression level under normal conditions. p53 can be activated by different signalling pathways under cellular stresses such as DNA damage, which results in cell cycle arrest, apoptosis or fading by enhancing the transcription of multiple downstream genes, maintaining cell genome integrity, and clearing damaged cells [21, 22]. As an example, lncRNA-p21 recruits ribonucleoprotein hnRNP-k to promote P21 transcription, a key molecule regulating the p53 signalling pathway. Downregulation of lincRNA-p21 causes losing control of G1/S checkpoints and leads to enhanced cell proliferation [23]. Furthermore, the mechanism of lncRNAs regulating microRNAs is that microRNAs (miRNAs) are related to the development of many diseases, including liver cancer [24]. miRNAs can bind to lncRNA sponges to inhibit gene expression and protein synthesis [25]; therefore, they affect the function of the cell, e.g., lncRNA XIST promotes HCC proliferation and inhibits apoptosis by regulating miR-139-5p/PDK1/AKT axis [26]. Finally, lncRNAs take part in signalling pathways and hormonal regulation. Evidence shows that liver cancer development is associated with the abnormal activation of signalling pathways. The role of lncRNAs in these signalling pathways is an essential part of the mechanism of liver cancer. Therefore, future studies on lncRNAs are also expected to find candidate drugs for the treatment of liver cancer. Studies have confirmed the roles of transforming growth factor beta (TGF-*β*), the AKT signalling pathway, and the Wnt signalling pathway in tumor development [27–29]. For example, TGF-*β* promotes liver cancer cell metastasis via lncRNA-ATB. Abnormal AKT signalling leads to increased expression of lncRNA PTTG3P and promotes proliferation and migration of hepatoma cells.

## 3. LncRNAs in HCC

### 3.1. Dysregulation of LncRNAs in HCC

LncRNAs play irreplaceable roles in the progression of HCC because lncRNAs are known to be involved in the regulation of tumor differentiation at the tumor node metastasis stage (TNM) and cell growth processes, including cell proliferation and apoptosis, invasion, and metastasis. LncRNAs play an irreplaceable role in the progression of HCC. The key biological functions of lncRNA are related to certain signalling pathways. LncRNA expression in liver cancer tissues is associated with clinicopathological features. Deregulated lncRNAs can be a novel biomarker for diagnosing or assessing treatment efficiencies. The dysregulation of lncRNA in HCC marks a disease spectrum and is proposed to be associated with liver cancer. Many oncogenes are known as targets for liver cancer-associated lncRNAs (Table 1). LncRNA participates in HCC processes by binding to oncogenes. In addition, lncRNA can also participate in HCC through regulatory signalling pathways, even though the underlying mechanisms are still unknown. Differential expression and potential functional roles of lncRNAs in HCC are essential.

### 3.2. Upregulation of LncRNAs in HCC

#### 3.2.1. HOTAIR

HOTAIR, a 2158 bp lncRNA, is encoded in the HOXC locus on chromosome 12q13.1 [30]. It has been found that HOTAIR is more highly expressed in HCC tissues than in paracancerous nontumor tissues. The increased expression of HOTAIR is associated with lymph node metastasis; therefore, the HOTAIR expression level is associated with lymph node metastasis. Thus, the high HOTAIR levels in LT patients indicate a significantly shorter recurrence-free survival. Patients with tumors and high HOTAIR gene expression levels have a higher risk of recurrence after hepatectomy [31]. It is reported that HOTAIR promotes cell proliferation, autophagy, and invasion and reduces the response of hepatoma cells to the apoptosis stimulator TNF-*α* and the chemotherapeutic drugs cisplatin and doxorubicin [31–33]. Further studies revealed that tumorigenesis is suppressed in HCC after silencing HOTAIR, which resulted in the activation of P16 and P14 signalling via increased miR-218 expression and decreasing Bmi-1 expression, respectively. It is suggested that HOTAIR expression is associated with liver tumor differentiation, metastasis, and early recurrence [34]. These findings suggest that HOTAIR plays an important role in the development of hepatocarcinoma, so HOTAIR is the possible target for the diagnosis and treatment of liver cancer.

#### 3.2.2. MALAT1

MALAT1 (metastasis-associated lung adenocarcinoma transcript 1) was initially discovered in human non-small-cell lung cancer (NSCLC). MALAT1 is the first identified metastasis-associated lncRNA, which is found upregulated in HCC cell lines and patients [35, 36]. Functionally, MALAT1 promotes proliferation, invasion, metastasis, chemosensitivity, and autophagy in HCC cells. MALAT1 is upregulated in HCC and associated with cell proliferation and migration by regulating Bax, bcl-2, bcl-xl, caspase-3, and caspase-8 [36]. Moreover, MALAT1 is thought to promote proliferation during liver regeneration through the stimulation of the Wnt/catenin pathway, which is negatively regulated by p53. Silencing MALAT1 reduces cell viability, migration, and invasion and increases the sensitivity of cells to apoptotic stimuli such as cisplatin and doxorubicin, TNF-alpha, and glucose-brain toxins [37]. Thus, MALAT1 is probably involved in tumor development and could be a new biomarker for predicting tumor recurrence after LT.

#### 3.2.3. HULC

HULC is the first identified lncRNA specifically upregulated in HCC [38]. Its coding sequence is located on chromosome 6p24.3 and is highly conserved among primates. In clinical tissues, HULC expression in HCC and hepatic colorectal metastasis samples is highly upregulated compared with that in the normal control. HULC expression is positively correlated with the Edmondson histological classification and the HBV/HBV X protein- (HBx-) positive state [39–41]. Similar results were detected in plasma samples from hepatocellular carcinoma [41]. HULC promotes lipogenesis and cell proliferation, induces apoptosis and enhanced epithelial-mesenchymal transformation (EMT), and also increases the risk of liver cancer development and metastasis [40]. Previous studies illustrated that HBx-mediated HULC upregulation promotes the increase of mRNA and protein levels in liver cancer by downregulating the tumor suppressor gene CDKN2C (p18). CDKN2C is thought to be a tumor suppressor gene that regulates cell cycle and plays a role in signal transduction pathways, including ATM/ATR and p53 pathways. These results indicated that HULC could be used as a potential biomarker for HCC diagnosis.

#### 3.2.4. PTTG3P

**Figure 3:**
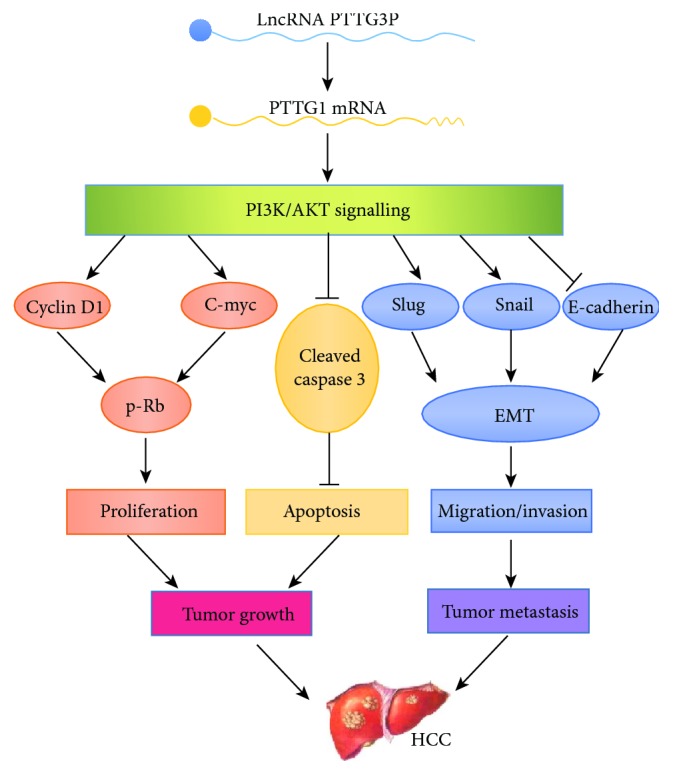
Functional diagram of lncRNA PTTG3P in HCC tumor growth and metastasis.

PTTG3P (pituitary tumor-transforming 3 pseudogene) is a novel lncRNA. Expression and localization of PTTG3P were analyzed using quantitative real-time polymerase chain reaction (qRTPCR) and in situ hybridization (ISH) in two patients with liver cancer [42]. It has been shown that the expression of PTTG3P in liver cancer is significantly increased. The upregulation of PTTG3P is positively correlated with a poor prognosis of liver cancer patients. PTTG3P promotes cell proliferation, inhibits apoptosis, and accelerates migration and invasion of HCC cells. Mechanically, PTTG3P is involved in tumor growth, while PTTG3P metastatic cascade promotes cells by the upregulation of PTTG1 and activation of the PI3K/AKT signalling pathway. Growth and metastasis follows, which in turn affect downstream signal transduction, through the regulation of cell cycle regulators and EMT-related factors as shown in Figure 3. Therefore, PTTG3P may be a potential target for the prevention and treatment of liver cancer [43].

#### 3.2.5. PVT1

LncRNA PVT1 is located at 8q24.21 [44]. Studies showed that PVT1 is highly expressed in HCC tissues and associated with the number and grade of tumors. PVT1 promotes tumor growth by accelerating cell proliferation and cell cycle progression and enhancing stem cell-related properties [45]. Functionally, PVT1 can increase NOP2 levels by enhancing the stability of the NOP2 protein, and the function of PVT1 is dependent on the presence of the NOP2 protein. The hPVT1/NOP2/cell cycle gene pathway is involved in promoting carcinogenesis, cell proliferation, and stem cell-like properties in HCC cells [14]. Furthermore, it has been reported that PVT1 promotes proliferation, invasion, and migration of liver cancer cells by modulating the mir-150/HIG2 axis [44]. Therefore, PVT1 can be used as a diagnosis marker for HCC.

### 3.3. Downregulation of LncRNAs in HCC

#### 3.3.1. MEG3

MEG3 is highly expressed in the human pituitary gland and is a maternal imprinting gene. The MEG3 gene is located at the imprinted DLK1-MEG3 locus on chromosome 14q32.3 in humans. MEG3 expression is observed in several types of cancer [46]. MEG3 was confirmed downregulated in HCC. MEG3 regulates proliferation and apoptosis in HCC cells [47]. Mechanistically, MEG3 improves the protein stability, increases the transcriptional activity of p53 in hepatocellular carcinoma cells, and influences the expression of p53 target genes. In liver cancer tissues, MEG3 is negatively correlated with UHRF1 that plays an important role in DNA methylation by recruiting DNA methyltransferase DNMT1 during DNA replication. In the same study, UHRF1 is identified as involved in the upstream regulation of MEG3 in liver cancer by regulating DNMT1 [47]. These results indicate that MEG3 is a tumor suppressor gene and can be considered as a biomarker for liver cancer.

#### 3.3.2. DREH

DREH is involved in HBx-mediated hepatocellular carcinoma. DREH and mouse homologous DREH were significantly downregulated in human HBV-related HCC tissues and HBx transgenic mice, respectively. DREH is a highly conserved lncRNA. The DREH reduction is significantly associated with poor survival in patients with liver cancer [48]. DREH is linked to the proliferation and metastasis of HBV-related HCC. A previous study revealed the negative correlation between Dreh expression and HBx and HBs [49]. Dreh is downregulated by HBx via the downregulation of vimentin, which results in the suppression of HCC growth and migration [48, 49]. These results indicate that DREH is a tumor suppressor in the development of HBx-related hepatocellular carcinoma and may be a new target for the treatment of HBV-related hepatocellular carcinoma.

#### 3.3.3. LET

“Low expression in the tumor,” or LET, is present at significantly reduced levels in HCC tumor tissues and is linked to metastasis [50]. LET influences the invasiveness and metastasis of HCC cells. LET is inhibited by histone deacetylase 3 (HDAC3). LET inhibition increases the stability of nuclear factor 90 (NF90), thus promoting hypoxia-induced invasion [50]. HDAC3 induced by hypoxia inhibits lncRNA-let by reducing the lncRNA-let promoter region regulation mediated by histone acetylation. Interestingly, downregulation of lncRNA-let is a key step in stabilizing the NF90 protein, leading to hypoxic-induced infiltration of cancer cells. In addition, the relationship between hypoxia, histone acetylation disorders, low lncRNA-let expression, and metastasis has been demonstrated in clinical HCC samples. These findings demonstrated the role of lncRNA-let as a regulator of hypoxia signal transduction and provide new methods for therapeutic intervention in cancer progression. Moreover, these findings also indicate that hypoxia can inhibit lncRNA-let expression by reducing the level of acetylation of histones H3 and H4 in its promoter region [50]. In addition, downregulation of lncRNA-let may affect the accumulation and stability of hif-1a mRNA under hypoxia.

## 4. Biological Roles of LncRNAs in HCC

### 4.1. Proliferation

To date, it has been proven that many lncRNAs maladjusted in hepatocellular carcinoma play a vital role in the growth of hepatocellular carcinoma in vitro or in vivo. It has been found that upregulated URHC promotes tumor and cell proliferation, which is directly related to poor prognosis in liver cancer tissues and cell lines. Further studies showed that URHC promotes cell proliferation by regulating ZAK protein during the activation of the ERK/MAPK signalling pathway [9]. Researchers found that H19 knockdown abolishes the tumorigenicity of HCC in vivo, and significantly, hypoxic recovery reduces growth independent of the adherent wall [51]. Moreover, the detection of the hepatoma cell line Bel7402 in vitro showed that silencing lncRNA HOTAIR could inhibit cell proliferation [31].

### 4.2. Apoptosis

Apoptosis is the gene-controlled autonomous and orderly death of cells. The decrease of apoptosis can promote the survival and accumulation of abnormal cells and lead to cancer development. It has been reported that lncRNA affects liver cancer by acting on apoptosis. The expression of uc002mbe.2 is lower in liver cancer cell tissues than in normal ones. The histone deacetylase inhibitor Trichostatin A (TSA) exerts an antitumor effect by promoting the apoptosis of liver cancer cells. Apoptosis induced by TSA is significantly inhibited by uc002mbe.2 knockdown [52]. Thus, uc002mbe.2 is very important in TSA-mediated hepatocyte apoptosis.

### 4.3. Invasion and Metastasis

The most crucial reasons for mortality and poor prognosis in patients with liver cancer are tumor metastasis and invasion, which are related to HCC both in vitro and in vivo. More and more evidences showed that lncRNA plays an important role in the invasion and metastasis of liver cancer. For example, lncRNA-related microvascular invasion in HCC (MVIH) are a class of lncRNA molecules that are highly expressed in liver cancer and are involved in angiogenesis. When the expression of these lncRNAs is high, the survival rate and prognosis are the opposite. Overexpression of MVIH in animal models promotes angiogenesis and facilitates tumor growth and metastasis. Furthermore, the expression of MVIH in liver cancer patients is significantly negatively correlated with the angiogenesis inhibitor PGK1, suggesting that MVIH promotes liver cancer metastasis by inhibiting the secretion of PGK1 [16]. In addition, recent studies indicated that lncRNA-ATB activated by TGF-*β* induces EMT and cell invasion in vitro, promoting the invasion of hepatocellular carcinoma cells [53].

## 5. Conclusions and Future Perspectives

**Figure 4:**
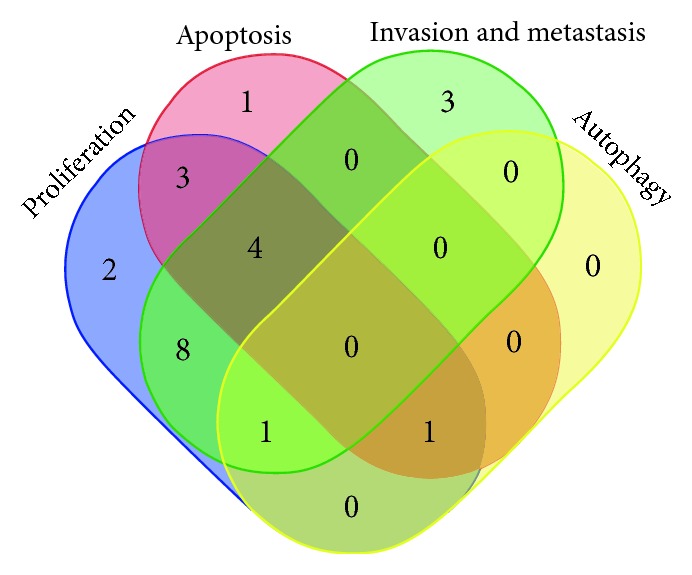
Informatics analysis of the biological functions of lncRNAs in HCC.

In conclusion, lncRNAs play important roles in the biological processes of the occurrence, development, metastasis, and recurrence of liver cancer, which impact on the treatment and prognosis of liver cancer. Furthermore, the dysregulation of liver cancer-associated lncRNA in tumor tissues is often associated with these biological processes. LncRNA dysregulation is associated with the progression and prognosis of liver cancer. Therefore, lncRNA should a candidate biomarker for the diagnosis, prognosis, recurrence prediction, and treatment of liver cancer. The characteristics of the biological functions related to HCC lncRNA enable researchers to have a more comprehensive understanding of the occurrence of liver cancer (Figure 4, Table 2). Further research on the mechanism of lncRNA involvement in the development and progression of liver cancer is conducive to clinical diagnosis and treatment. Although a small number of lncRNAs have been studied in liver cancer, a large part of them needs to be further discovered. Therefore, further research is needed on the role and mechanism of liver-specific lncRNA in the progression of liver cancer. The knowledge of the function of lncRNA in liver cancer development is increasing, which is laying the foundation for the design of new treatment methods for liver cancer.

## Figures and Tables

**Table 1:** Dysregulated long noncoding RNAs (lncRNAs) associated with HCC.

LncRNAs	Expression	Affected target genes and pathways	Affected clinicopathological characteristics of HCC	References
HOTAIR	Upregulated	HOXD/VEGF/MMP-9/PRC2/H3K27/rbm38/Bmi-1/P14/P16	TNM stage, distant metastasis	[31–34]
HULC	Upregulated	P18/PRKACB/CREB	TNM stage, intrahepatic metastases	[38, 39, 54]
H19	Upregulated	Cdc25A/E2F1/hnRNP U/PCAF/DMC/ZEB1/2		[51, 55]
URHC	Upregulated	ZAK	Tumor size, tumor number	[9]
ROR	Upregulated	TGF-*β*/PDK1/P53		[56]
PVT1	Upregulated	TGF-*β*/NOP2	AFP level, tumor size, tumor number, tumor stage	[14, 57]
PTTG3P	Upregulated	PPTG1/AKT signalling	Tumor size, TNM stage	[43]
XIST	Upregulated	miR-139-5p/PDK1/AKT signalling	Tumor size	[26]
DBH-AS1	Upregulated	P53/ERK/MAPK signalling	HBsAg, tumor size	[58]
MEG3	Downregulated	UHRF1/P53	Tumor size, Edmondson grade	[47, 59]
DREH	Downregulated	HBx/vimentin	Tumor size, HBsAg	[48, 49]
PTENP1	Downregulated	miR-17/miR-19b/miR-20a/AKT/PI3K signalling	Tumor size, TNM stage	[60]
LET	Downregulated	P53/NF90/HIF-1*α*		[50]
uc002mbe.2	Downregulated	TAS	Tumor size	[52]

**Table 2:** Statistical analysis of lncRNAs and tumor biological functions.

Biological functions	Number	LncRNAs	References
Apoptosis, invasion, metastasis, and proliferation	4	PTTG3P	[43]
		DREH	[49]
		ANRIL	[61]
		HULC	[53]

Apoptosis, autophagy, and proliferation	1	PTENP1	[60]

Autophagy, invasion, metastasis, and proliferation	1	HOTAIR	[33]

Apoptosis and proliferation	3	uc002mbe.2	[52]
		DBH-AS1	[58]
		MEG3	[59]

Invasion, metastasis, and proliferation	8	CCAT1	[62]
		HOTTIP	[63]
		AFAP1-AS1	[64]
		UCA1	[65]
		H19	[55]
		XIST	[26]
		ZEB1-AS1	[66]
		HEIH	[67]

Proliferation	2	PVT1	[57]
		ROR	[56]

Apoptosis	1	URHC	[9]

Invasion and metastasis	3	HBx-LINE1	[68]
		LET	[50]
		ATB′	[69]
